# Poliosis as a clinical sign of melanoma arising on a congenital nevus^؆^

**DOI:** 10.1016/j.abd.2026.501340

**Published:** 2026-04-22

**Authors:** Silvestre Matínez García, Alejandro Arroyo Córdoba, Irene López Riquelme

**Affiliations:** Department of Dermatology, Hospital Regional Universitario de Málaga, Málaga, Spain

Dear Editor,

Poliosis, a localized patch of white hair, may rarely signal underlying melanoma, particularly when appearing over a melanocytic lesion.^1^ We report a case of poliosis over the scalp associated with melanoma arising on a congenital nevus and provide a brief literature review.

A 30-year-old man with no relevant medical history presented with a painful, palpable nodule on the right parotid region, evolving over five months. Histological evaluation of the lesion confirmed intraparotid lymph node metastasis of melanoma. Clinical examination revealed a congenital melanocytic nevus measuring 8 × 6 cm in the right temporal scalp, with recent enlargement and a central area of white hair (Fig. 1). Partial biopsy confirmed melanoma arising in a congenital nevus.

**Fig. 1:**
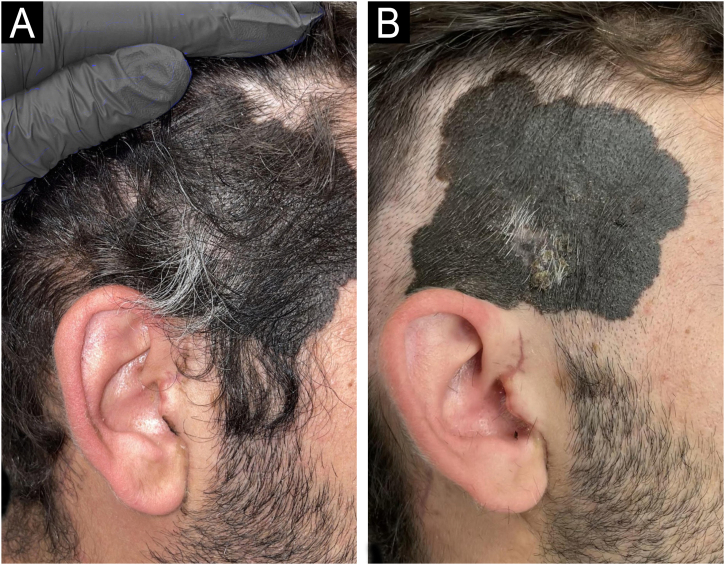
(A) Initial presentation showing localized white hair (poliosis) within a congenital nevus on the right temporal scalp. (B) Same lesion after partial shaving reveals central ulceration and color heterogeneity; histopathology confirmed invasive melanoma arising within the nevus.

The patient underwent complete excision of the lesion, total parotidectomy with facial nerve preservation, and right posterior cervical lymphadenectomy. A staging body CT scan excluded distant metastasis (M0). Histopathology showed invasive melanoma on a congenital nevus (Breslow 1.8 mm, Clark level III, ulceration, regression, 2 mitoses/mm^2^, BRAF V600E positive) (Fig. 2), with 4 of 37 lymph nodes involved, one with extracapsular extension, corresponding to stage IIIC (AJCC 8). Adjuvant immunotherapy with pembrolizumab was administered for one year, and after 15-months of follow-up, the patient remains free of metastasis.

**Fig. 2:**
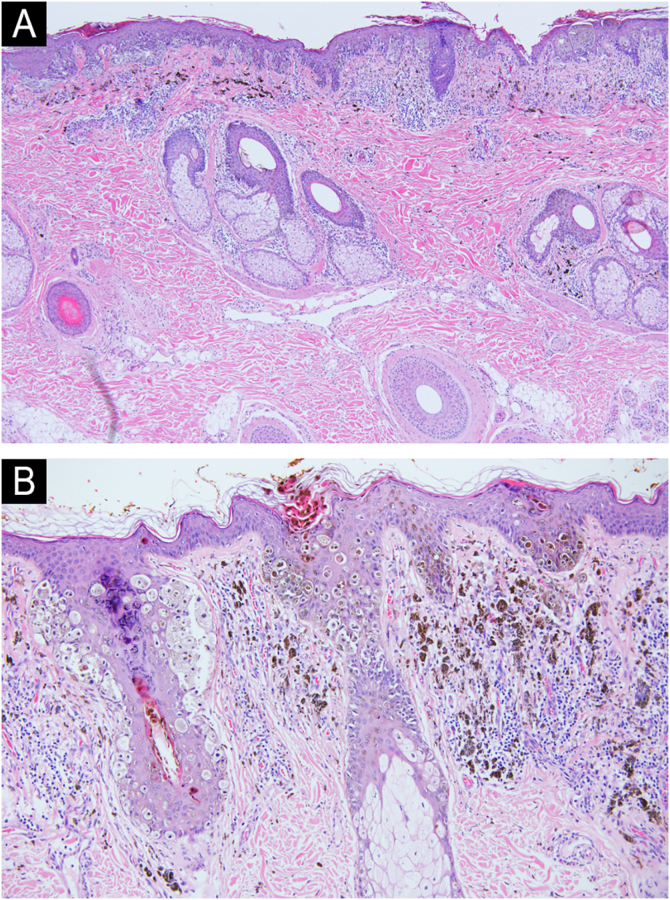
(A) Hematoxylin & eosin stain, original magnification ×10. Continuous lentiginous growth of atypical melanocytes with formation of large nests. Melanocytic infiltration of the deep dermis and adnexal structures is also observed, a characteristic feature of congenital nevi. (B) Hematoxylin & eosin stain, original magnification ×40. Large, atypical melanocytes showing pagetoid spread and infiltration of the hair follicle.

Poliosis has been associated with a broad spectrum of conditions, including autoimmune diseases (e.g., vitiligo, Vogt-Koyanagi-Harada, alopecia areata), inflammatory dermatoses, drug reactions (e.g., prostaglandin analogues, checkpoint inhibitors), and both benign and malignant tumors.^2^, ^3^ In melanoma, poliosis may reflect the destruction of follicular melanocytes through immune-mediated mechanisms. Molecular mimicry or shared antigens between melanoma cells and follicular melanocytes may explain this phenomenon.

We identified 8 previously reported cases in the literature directly linking circumscribed poliosis to melanoma (Table 1, Supplementary Table S1 and Fig. S1). Among these cases, most occurred in men and involved the scalp. Two cases described poliosis of the eyelashes associated with ocular melanomas ‒ one conjunctival and one orbital ‒ highlighting the relevance of poliosis in this site as a potential clue to intraocular disease. Additionally, in two cases, poliosis appeared at locations distant from the primary tumor, including the scalp and eyelashes, while the melanoma was located on the lower limb. This suggests that poliosis may occasionally act as a clinical marker of metastatic spread from a distant primary melanoma.

**Table 1:** Clinical and histopathological characteristics of reported melanoma cases associated with poliosis.

Author/Year	Type of Study	Sex	Age	Evolution	Medical history	Melanoma	Location Melanoma	Histological examination	Poliosis	Location Poliosis	Sentinel lymph node	Dx
Dunn CL 1995	Case report	Man	42	2 years	No medical history	Superficial spreading melanoma	Scalp	Breslow 1.3 mm, regression	In melanoma	Scalp	Unrealized	Localized disease
de Alba Campomanes AG 2008	Case report	Woman	71	2 months	Melanoma 4 years before	Conjunctival	Conjunctival	Not appear	In melanoma	Eyelashes	Unrealized	Localized disease
Alsuhaibani AH 2011	Case report	Man	60	3 months	No medical history	Orbital	Orbital	Not appear	In melanoma	Eyelashes	Unrealized	Localized disease
Yeo L 2015	Concise report	Man	28	1 year	No medical history	Superficial spreading melanoma	Scalp	Breslow 0.8 mm, regression, no ulcerate, 4 mitoses per mm^2^	In melanoma	Scalp	Negative	Localized disease
Fernández-Díaz MR 2019	Medicine in images	Man	74	1 month	Melanoma 3 years before	Superficial spreading melanoma	Right leg	Not appear	Not adjacent	Eyelashes, eyebrows and scalp	Lymph node positive	Disseminated disease
Schollenberger MD 2019	Case report	Woman	31	1 year	No medical history	Superficial spreading melanoma	Scalp	Pigmented melanophages and early dermal fibrosis; no melanocytic proliferation	In melanoma	Scalp	Lymph node positive	Lymph node disease
Burzi L. 2021	Letters to the Editor	Woman	65	2 meses	No medical history	Amelanotic	Plantar	Not appear	Not adjacent	Eyelashes	Lymph node positive	Disseminated disease
Karch JL 2023	Case report	Man	44	Since birth	No medical history	Ex blue nevus	Scalp	Breslow 16 mm	In melanoma	Scalp	Lymph node positive	Lymph node disease
Our case 2025		Man	30	6 months	No medical history	Congenital naevus	Scalp	Breslow 1.8 mm, ulceration, regression, 2 mitoses per mm^2^, BRAF-positive	In melanoma	Scalp	Lymph node positive	Lymph node disease

Histological features frequently included areas of regression and fibrosis in association with poliosis. Though regression in melanoma remains a debated prognostic factor, recent meta-analyses suggest it may be associated with improved survival.^4^ Nevertheless, in this small series, 5 of 9 patients showed nodal or systemic involvement, underscoring the importance of prompt recognition and staging. With these findings, poliosis may represent a late-stage sign, potentially associated with a poorer prognosis in melanoma.

Scalp melanomas comprise 3%–5% of cutaneous melanomas but are associated with delayed diagnosis and worse outcomes. Their frequent amelanotic presentation contributes to diagnostic difficulty.^5^ In this context, the emergence of localized poliosis over a nevus, particularly in adults, should prompt immediate dermoscopic evaluation and consideration of biopsy.

In conclusion, we present a rare case of melanoma arising in a congenital scalp nevus associated with circumscribed poliosis. Clinicians should consider recent onset of poliosis over melanocytic lesions as a potential clinical sign of melanoma requiring early investigation. Special attention should be paid to poliosis of the eyelashes, which may reflect not only ocular melanoma but also metastatic disease from a distant primary tumor.

## ORCID ID

Silvestre Martínez García: 0000-0002-2399-5325

Alejandro Arroyo Córdoba: 0009-0000-5349-8592

Irene López Riquelme: 0000-0002-6488-4381

## Financial support

None declared.

## Authors' contributions

Silvestre Martínez García: Conceptualization; methodology; writing-original draft preparation.

Alejandro Arroyo Córdoba: Writing-final draft preparation; editing and validation.

Irene López Riquelme: Supervision; critical review and editing.

## Research data availability

Does not apply.

## Conflicts of interest

None declared.

## References

[1] Sleiman R., Kurban M., Succaria F., Abbas O. (2013). Poliosis circumscripta: overview and underlying causes. J Am Acad Dermatol..

[2] Jalalat S.Z., Kelsoe J.R., Cohen P.R. (2014). Alopecia areata with white hair regrowth: case report and review of poliosis. Dermatol Online J..

[3] Burzi L., Alessandrini A.M., Quaglino P., Piraccini B.M., Dika E., Ribero S. (2021). Cutaneous events associated with immunotherapy of melanoma: A review. J Clin Med..

[4] Ribero S., Moscarella E., Ferrara G., Piana S., Argenziano G., Longo C. (2016). Regression in cutaneous melanoma: a comprehensive review from diagnosis to prognosis. J Eur Acad Dermatol Venereol..

[5] Garbayo-Salmons P., Sàbat Santandreu M., Fernández-Chico N., Luelmo Aguilar J. (2022). Scalp melanoma: clinical and histopathological findings. Actas Dermosifiliogr..

