# Supplementary material for: Poliosis as a clinical sign of melanoma arising on a congenital nevus
Source: An Bras Dermatol. 2026 Apr 22;101(3):501340. doi: 10.1016/j.abd.2026.501340 (PMC13123486; doi:10.1016/j.abd.2026.501340)
Supplement: Supplementary file 1 [file mmc1.docx]

ABD-D-25-00537_Supplementary Material

**Supplementary Figure 1** PRISMA flow diagram illustrating the study selection process.


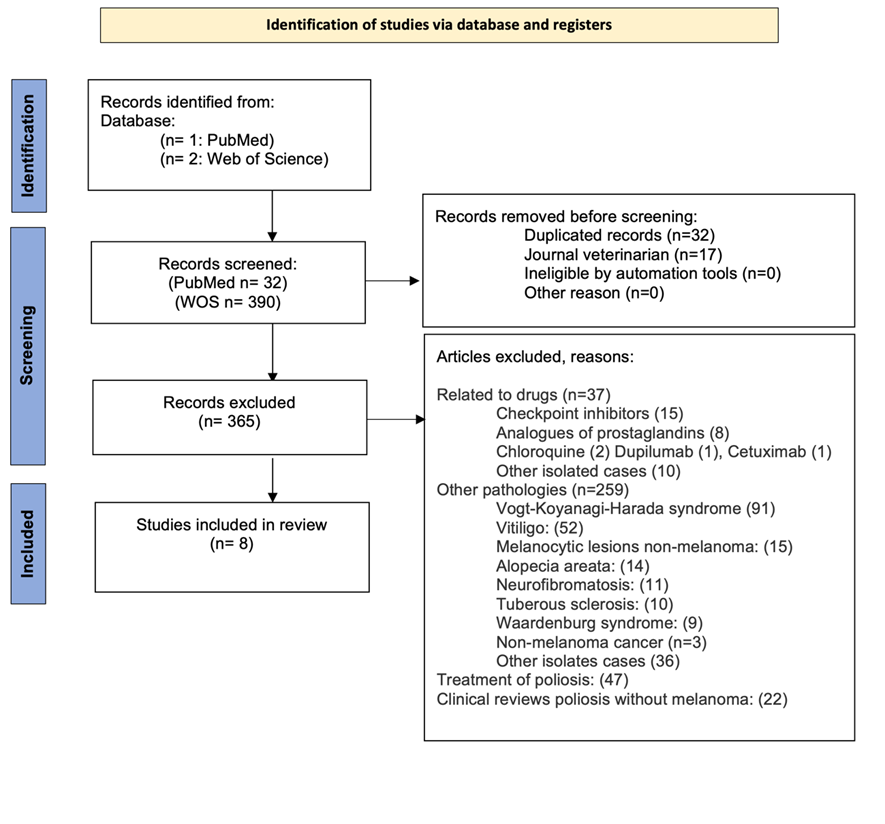


**Supplementary Table 1** Bibliographic references of the eight reviewed articles describing melanoma cases associated with poliosis.

| 1. | Dunn CL. Melanoma of the scalp presenting as poliosis circumscripta. *Archives of Dermatology*. 1995;131(5):618-619. doi:10.1001/archderm.131.5.618 |
| --- | --- |
| 2. | de Alba Campomanes AG. Poliosis as a Manifestation of Conjunctival Melanoma. *Archives of Ophthalmology*. 2008;126(7):1006. doi:10.1001/archopht.126.7.1006 |
| 3. | Alsuhaibani AH. Primary Orbital Melanoma with Poliosis and a Palpable Mass. *Archives of Ophthalmology*. 2011;129(10):1382. doi:10.1001/archophthalmol.2011.302 |
| 4. | Yeo L, Husain E, Rajpara S. Poliosis circumscripta unmasking a scalp melanoma. *Clinical and Experimental Dermatology*. 2015;40(8):872-874. doi:10.1111/ced.12523 |
| 5. | Fernández-Díaz MR, Trigo-Rodriguez M, Faro-Miguez N, Aneiros-Fernández J, Parejo-Morón AI. Metastatic melanoma-asociated poliosis. *Revista Clinica Espanola*. 2019;219(7):411-412. doi:10.1016/j.rce.2018.07.004 |
| 6. | Schollenberger MD, Stein JE, Taube JM, Lipson EJ. Poliosis Circumscripta: A Mark of Melanoma. *The American Journal of Medicine*. 2019;132(12):1417-1418. doi:10.1016/j.amjmed.2019.05.042 |
| 7. | Burzi L, Parietti M, Agostini A, et al. Eyelashes poliosis as first sign of metastatic melanoma. *Journal of the European Academy of Dermatology and Venereology*. 2021;35(7). doi:10.1111/jdv.17215 |
| 8. | Karch JL, Davis MJ, Momtahen S, Simmons BJ. Subcutaneous Nodule with Poliosis: An Unusual Presentation of Melanoma Ex Blue Nevus. *Dermatologic Surgery*. Published online September 18, 2023. doi:10.1097/DSS.0000000000003951 |
